# A single-cell and multi-platform spatial atlas of the human pancreas resolves a transformation-associated epithelial axis and a recurrent boundary-organized tumour microenvironment

**DOI:** 10.64898/2026.07.31.742073

**Published:** 2026-08-03

**Authors:** Kaili Liu, Hua Zhong, Rahhul S. Elangovan, Yuanhong Sun, Lin Wang, Abigae P. Williams, Trisha I. Valerio, Coline Furrer, Jacob P. Adams, Ashley R. Hoover, Wei R. Chen

## Abstract

Most pancreatic ductal adenocarcinoma (PDAC) single-cell and spatial studies analyze one cohort or platform, obscuring recurrent biology. We assembled a human pancreas single-cell and single-nucleus reference of 1,186,130 cells from 19 studies and interpreted 176 Visium sections comprising 458,877 spots across non-diseased pancreas, chronic pancreatitis, PanIN, IPMN, primary PDAC and metastasis. Marker-supported labels were used after two RNA-based copy-number callers failed known-diploid controls. BANKSY domains, two reference-mapping methods and sample-level analyses resolved a cross-sectional epithelial axis extending from acinar-rich to malignant tissue. Five trajectory algorithms recovered similar ordering on a shared embedding; their consensus was interpreted as transformation-associated, not temporal or clonal. Malignant regions were globally segregated from fibroblast and myeloid compartments. Signed-distance analysis refined this pattern into a malignant core, a CAF/myeloid surround beginning at the tumour boundary and a more distal lymphoid compartment. Candidate extracellular-matrix communication, led by COLLAGEN, LAMININ and FN1, concentrated at the interface. Changes were reproduced in six patient-matched Normal-tumour pairs using exact patient-level tests. Visium HD resolved the same organization at single-cell resolution and showed that 8-um bins distorted immune-adjacency estimates. Xenium also revealed recurrent neighbourhoods but sample-specific stromal boundaries. We provide a confound-aware framework for identifying recurrent epithelial and microenvironmental organization in PDAC.

## Full Text Availability

The license terms selected by the author(s) for this preprint version do not permit archiving in PMC. The full text is available from the preprint server.

